# Geological constraints on dynamic changes of fluid pressure in seismic cycles

**DOI:** 10.1038/s41598-022-19083-x

**Published:** 2022-08-30

**Authors:** Takahiro Hosokawa, Yoshitaka Hashimoto

**Affiliations:** Department of Global Environment and Disaster Prevention, Faculty of Science and Technology, Kochi University, Akebonocho 2-5-1, Kochi, 780-8520 Japan

**Keywords:** Structural geology, Tectonics, Geophysics

## Abstract

Fluid pressure along faults plays a significant role in fault behaviors in seismic cycles in subduction zones. When a thermal pressurization event occurs, the fluid pressure rises; conversely, when a fault-valve behavior event occurs, the fluid pressure falls. The stress state changes with seismic cycles from a reverse fault regime to a normal fault regime, as observed in both geophysical observations and geological records. Fluid pressure has been estimated for both modern accretionary prisms and exhumed accretionary complexes. However, changes in fluid pressure on seismogenic faults have not been connected to seismic cycles. Here, we quantitatively show the dynamic change in fluid pressure in a seismogenic fault with geological evidence from an exhumed accretionary complex. We found extensional veins related to seismogenic fault records that exchanged stress states the during seismic cycles. We also constrained the fluid pressure quantitatively, both at an increasing stage during an event and at a decreasing stage after an event. In this procedure, we propose new methods to constrain the magnitude of vertical stress and rock tensile strength.

## Introduction

Variations in fluid pressure along seismogenic faults are important in understanding earthquake mechanisms^1^. The shear strength along a seismogenic fault changes with fluid pressure during seismic cycles, and the variations in the shear strength are time-dependent^1^. Recent experimental studies have also suggested that fluid pressure can affect the frictional stability of fault zones^2,3^.

There are two opposing models for the shift in fluid pressure caused by earthquakes. One is the thermal pressurization model, in which the fluid pressure rises due to frictional heating during a seismic slip. The increase in fluid pressure reduces the shear strength and promotes slip along a seismogenic fault^4–6^. The other is the fault-valve model, in which the fluid pressure decreases after an earthquake due to fluid escape along newly developed fractures or faults along a subducting plate interface, enhancing the shear strength along the fault. It has been shown that variations in shear strength due to changes in fluid pressure during an earthquake cycle, as well as stress changes, may be important in controlling the onset and recurrence of rupture on seismogenic faults along subduction plate interfaces^7^. Quantitative constraints on fluid pressure variations are therefore important because these two conflicting models can coexist at different timescales during an event, potentially leading to a better understanding of seismic mechanisms and recurrence timescales.

The change in the stress state from reverse to normal fault stress regimes was observed during the Tohoku earthquake^8,9^. The stress change from the reverse to normal fault stress regime occurred at least one day after the Tohoku earthquake^8^. A geophysical study has reported that the recovery of the stress state to almost the same stress state as that of the pre-earthquake period took six years following the Tohoku earthquake^10^. Similar changes in the stress state were also identified in on-land accretionary complexes and in the cores of the Taiwan Chelung-pu Fault Drilling Project (TCDP) using a micro-fault inverse method^11,12^. However, it is difficult to geologically estimate the time scale of the changes in the stress state.

In a previous study, a poro-elastic model was used to constrain the fluid pressure decrease during seismic cycles, as demonstrated by the extensional veins of the Nobeoka thrust in southwest Japan^13^. However, because the study only focused on extensional veins formed during normal fault stress regime, it did not fully capture the change in fluid pressure during seismic cycles.

In this study, we focus on the underplating thrust zone in a mélange zone within an exhumed accretionary complex. The mélange adjacent to the thrust zone has extensional veins showing network texture, which is related to the activity of the underplating thrust zone because of their occurrences^14^. We examined the change in the stress state with seismic cycles using mixed Bingham statistics^15,16^ for extensional veins. Then, the rock failure theory^13,17^ and fluid pressure obtained from fluid inclusion microthermometry for the veins^18^ instead of the poro-elastic model^13^ were employed to constrain the change in fluid pressure during seismic slip and after a seismic event in seismogenic zones along the subduction plate interface.

## Geological setting

**Figure 1:**
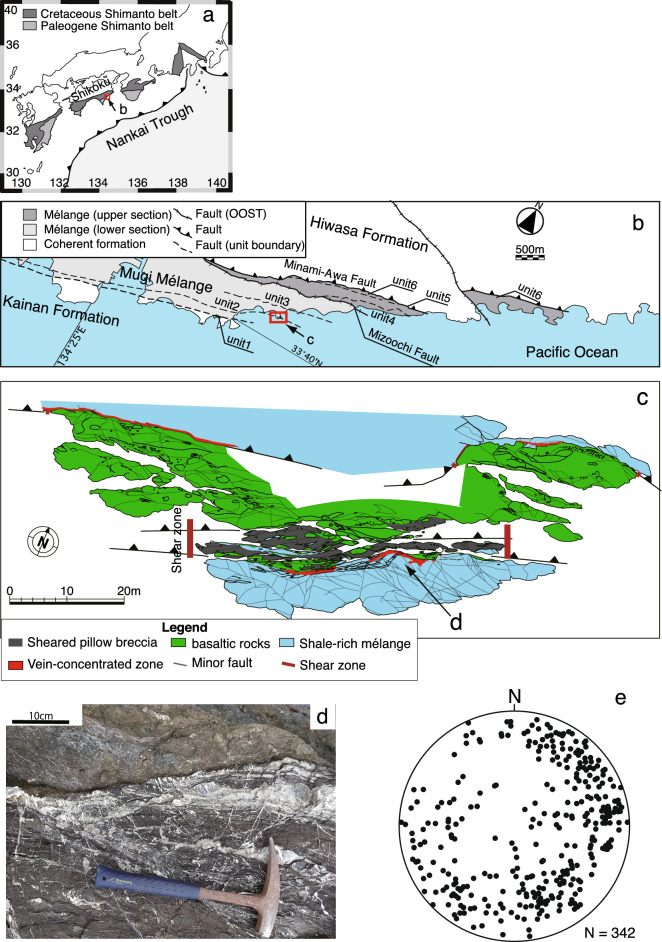
Geological setting of the study area. (**a**) Distribution of the Shimanto Belt in southwest Japan. (**b**) A regional geological map of the Mugi mélange, the Shimanto Belt modified from a previous study^20^. Upper and lower sections are divided by the Mizouchi Fault. The location of (**b**) is shown by an arrow in (**a**,**c**) A geological map of the study area modified from a previous study^14^. The shear zone is represented by vertical thick lines. The distribution of the network veins is indicated by the red area just south of the shear zone within the mélange. The location of the (**c**) is shown in (**b**) with an arrow. (**d**) A photograph of the network of the extensional veins. The location of the photograph is shown in (**c**) with an arrow. (**e**) The poles of the extensional veins plotted in a lower semi-sphere equal area stereonet. Maps (**a**–**c**) were created by Adobe Illustrator 26.3.1 (https://www.adobe.com/jp/products/illustrator.html).

The study area is an exhumed accretionary complex, the Mugi mélange in the Late Cretaceous to Early Paleogene Shimanto accretionary complex, southwest Japan^19,20^ (Fig. 1a). The Mugi mélange is composed of tectonic mélanges, mainly sandstone blocks and shale matrices, with traces of basalt, chert, tuff, and red shale. Oceanic materials represent the ocean floor stratigraphy, showing a map-scale duplex structure indicating that the Mugi mélange was formed by underplating^19,20^. The Mugi mélange is divided into lower and upper sections, with maximum paleotemperatures of 130–150 °C and 170–200 °C, respectively, separated by an out-of-sequence thrust^19,21,22^ (Fig. 1b). The lower section contains a sequence of three thrust imbricates (Units 1–3). The depositional age is approximately 60.5–62.3 Ma^20^. In addition, pseudotachylytes have been observed in the upper unit of the Mugi mélange, which provides strong evidence that the mélange was located within the seismogenic zone around a subduction plate interface^21,23^.

The fault zone we studied is related to underplating, comprising cataclasites and ultracataclasites mainly of basalts and a small number of cherts that steeply dip to the south or north and strike east-northeast direction^19^ (Fig. 1c). The average strike and dip of the shear zone (major shear surface) is N68° E 58° S. The thickness of the cataclasites and ultracataclasites (shear zone in Fig. 1c) ranges from about 3–7 m, and the total thickness of the fault zone, including the fractured damage zone, is about 20 m (Fig. 1c). Geological evidence of thermal pressurization, such as fluidization and injection of comminuted material, dilatational brecciation in extensional jogs, and the stretching of fluid inclusions in calcite veins, has been identified in the ultracataclasites in the fault zone^24–26^, suggesting that the shear zone represents a fossil seismic fault. Riedel shears within the shear zone indicate a dextral sense of shear in the current setting (Supplementary Fig. S1). The dextral sense of shears contradicts plate reconstruction showing a sinistral lateral convergence component in East Asia after the Late Cretaceous–Paleogene^27^. Therefore, the inconsistency in the sense of shear suggests that the shear zone is inverted and that there is possibly a mélange adjacent to the shear zone facing south.

The mélange immediately above the fault zone is composed of terrigenous sandstone and shale matrices. The extensional veins concentrate the boundary between the mélange and the shear zone, but only on the mélange side, and show a network texture (Fig. 1d). Because network veins grew solely along the shear zone, their occurrence must be linked to underplating after the mélange formation^14^. The thickness of the zone with network veins is approximately 20 cm. Fluid inclusion microthermometry for the network veins revealed fluid temperatures of 135(+ 10/− 20)–245 °C and pressures ranging 107(+ 8/− 4.1)–149.4 MPa^18^. The network veins formed after the mélange formation because they cut the mélange fabrics. Each vein in the network crosscuts with others without any specific orientation of veins, suggesting that multiple stress states were repeatedly exchanged (Fig. 1e). The microstructures of the vein minerals represent blocky and dendritic textures, suggesting that the network veins were formed by rapid precipitation from an oversaturated fluid^28,29^.

### Estimations of paleo-stress and driving fluid pressure

**Figure 2:**
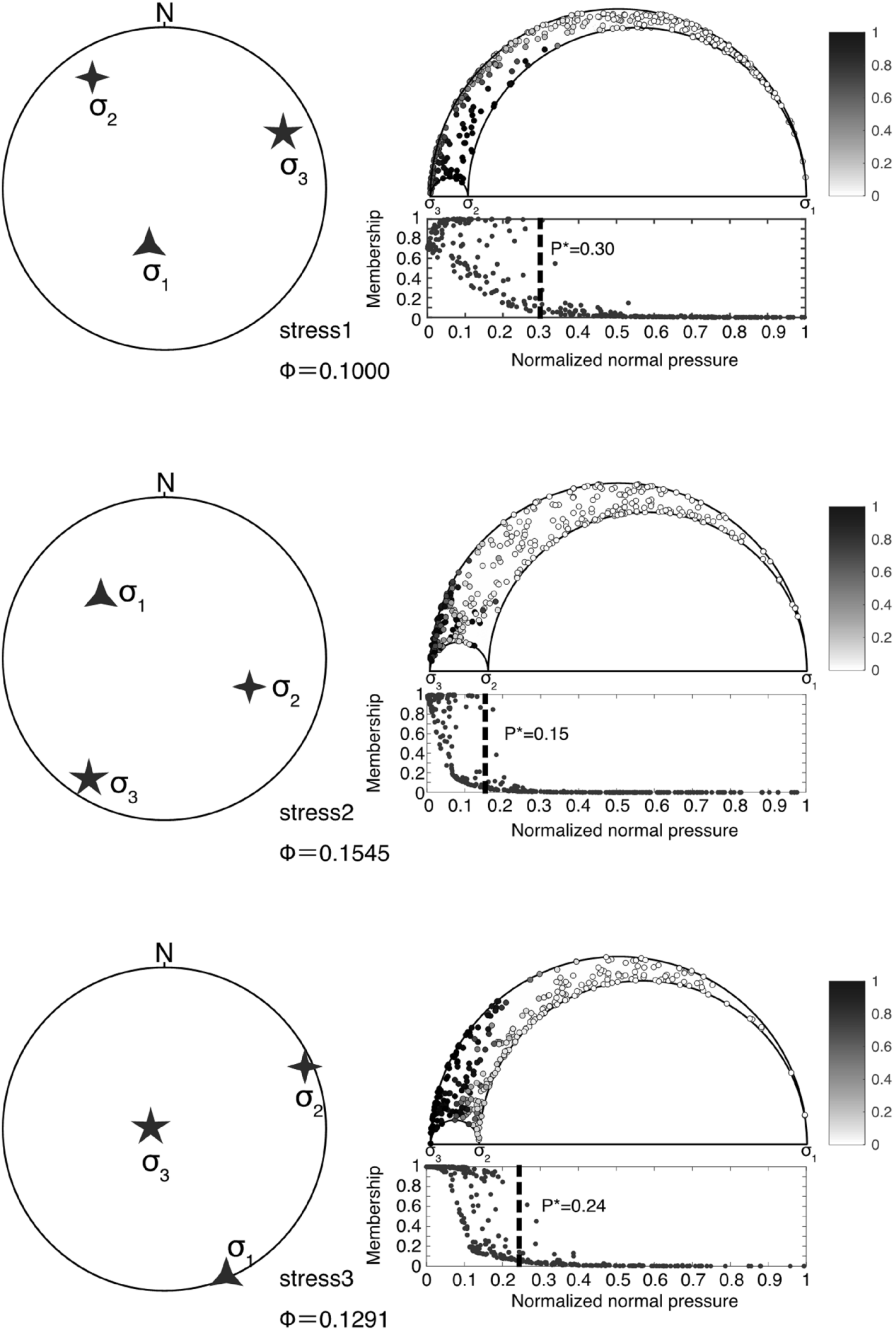
Inverted stress state after the rotation. The orientations of the principal stress axes for each stress state are shown in lower-hemisphere equal area stereonets in the left. A stress ratio ($\Phi$) is also shown. Normalized normal and shear stresses are plotted as small circles in Mohr’s circles for each stress state on the right. The gray color in the circles indicates membership (the probability that each extensional vein belongs to each stress state). Color bars for the membership are represented in the upper right. The relationship between the normal stress and membership is exhibited below each of Mohr’s circles. Membership decreases with the increase in normal stress, with a probability. Driving pressure (${P}^{*}$) equals the normal stress at which the member is zero.

We used mixed Bingham statistics^15,16^ to predict the stress state and driving fluid pressure (${P}^{*}$) for the network veins (see the Methods section). As a result, three stress states, Stress 1, 2, and 3, were identified, and ${P}^{*}$ for each stress state were estimated (Fig. 2 and Supplementary Fig. S2). For each stress state, the dip azimuth and dip angle of the maximum, intermediate, and minimum principal stresses were (174.9°, 9.2°), (298.5°, 73.7°), and (82.7°, 13.4°) for Stress 1; (210.2°, 69.8°), (304.6°, 1.6°), and (35.2°, 20.1°) for Stress 2; and (335.4°, 53.6°), (71.7°, 4.6°), and (165.1°, 36.0°) for Stress 3 (Supplementary Table S1). The stress ratios (Φ) (see the “Methods” section) for each stress state were 0.1000, 0.1545, and 0.1291, respectively. Stress 1 shows a strike-slip fault stress regime with sub-horizontal ${\sigma }_{1}$ in the NS direction and sub-horizontal ${\sigma }_{3}$ in the EW direction. Stress 2 represents a normal fault stress regime with subvertical ${\sigma }_{1}$ and sub-horizontal ${\sigma }_{3}$ in the NE–SW direction. Stress 3 exhibits a normal fault stress regime with sub-vertical ${\sigma }_{1}$ and sub-horizontal ${\sigma }_{3}$ in the NNW–SSE direction. In addition, the ${P}^{*}$ values for each stress state were 0.30, 0.15, and 0.24, respectively. ${P}^{*}$ is defined as the maximum fluid overpressure normalized by the differential stress for the extension veins, and it varies between 0 and 1^13^. A ${P}^{*}$ of 0 indicates that the minimum fluid pressure during extensional vein formation is equivalent to the rock failure theory conditions^17^. Our results show ${P}^{*}$ values that are all greater than 0, ranging from 0.15 to 0.30. This suggests that the extensional veins formed with an overpressure fluid larger than that predicted by the rock failure theory^17^ in the mélange just above the fault zone (see the “Methods” section).

### Change in stress state with seismic cycles

The shear zone was assumed as horizontal to the decollement plane at the time the extensional veins were developed because no thermal gap at the shear zone was observed; this suggests that the shear zone was parallel to the horizontal thermal structure^30^. This is the same interpretation as that for the Minami-awa fault with pseudotachylytes in the Mugi mélange^21^. Therefore, the shear plane must be rotated horizontally to estimate the stress state in the event. Aaccrodingly, the estimated principal stress orientations were rotated by 58° to the south with a rotation axis of N68° E based on the average strike and dip of the fault zone (N68° E 58° S). As a result of rotation, the dip azimuth and dip angle of the maximum, intermediate, and minimum principal stresses were (196.2°, 62.3°), (327.1°, 19.0°), and (64.0°,19.4°) for Stress 1, (316.2°, 42.6°), (108.3°, 43.9°), and (212.6°, 14.4°) for Stress 2, and (156.5°, 4.4°), (66.0°, 5.6°), and (284.1°, 82.9°) for Stress 3, respectively (Fig. 2). Stress 1 shows a normal fault stress regime with sub-vertical ${\sigma }_{1}$ and a WSW–ENE sub-horizontal ${\sigma }_{3}$. Stress 2 indicates a non-Andersonian stress regime with principal stresses, NW, intermediately plunged ${\sigma }_{1}$, NE, intermediately plunged ${\sigma }_{2}$ and SW sub-horizontal ${\sigma }_{3}$. Stress 3 is a reverse fault stress regime with NNW–SSE sub-horizontal ${\sigma }_{1}$ and sub-vertical ${\sigma }_{3}$. Although Stress 2 shows a non-Andersonian stress, reverse and normal fault stress regimes (Stress 1 and Stress 3) were obtained from the network veins among the three stress states. This is consistent with the results of previous studies on on-land accretionary complexes and the TCDP fault core^11,12^. The cross-cutting relationship of the extensional veins at the outcrop scale suggests that the extensional veins were repeatedly formed at the same depth under different stress regimes, as previously described. Therefore, the results of the stress inversion and outcrop observations imply that this fault zone records stress exchanges during seismic cycles.

### Constraints of depth and rock tensile strength

We focused on extensional veins formed in the normal and reverse fault stress regimes to constrain fluid pressure variations during seismic cycles. For that, the depth of vein development and rock tensile strength need to be constrained.

**Figure 3:**
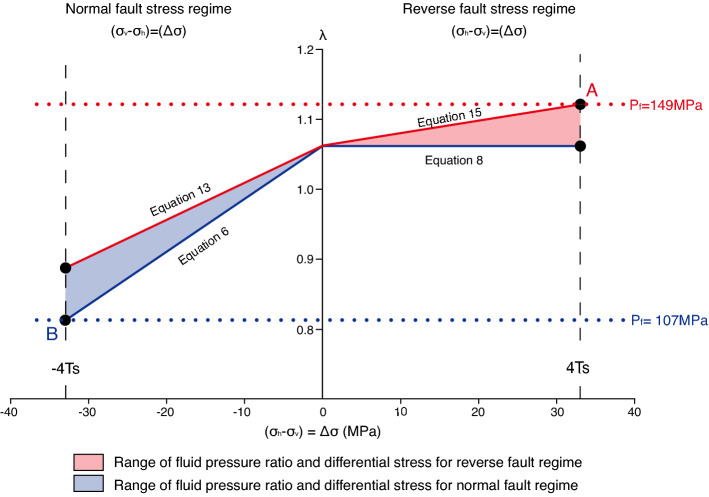
Relationship between the fluid pressure ratio ($\lambda$) and differential stress ($\Delta \sigma$). Differential stress equals the differences between horizontal stress (${\sigma }_{h}$) and vertical stress (${\sigma }_{v}$). Positive and negative differential stresses correspond to reverse and normal fault stress regimes. Lines from Eqs. (6), (8), (13), and (15) are shown for the constrained fluid pressure variations. Points A and B at 4${T}_{s}$ and ${-4T}_{s}$ indicate the maximum and minimum fluid pressures in the range. The maximum and minimum fluid pressures from fluid inclusion analysis are shown at points A and B.

Firstly, we used a $\lambda$–$\Delta \sigma$ diagram ($\lambda$ is the fluid pressure ratio, $\lambda ={P}_{f}/{\sigma }_{v}$) to constrain the fluid pressure ratio for extensional veins^7,31,32^. In the diagram, negative and positive $\Delta \sigma$ values represent the normal and reverse stress regimes, respectively (Fig. 3). After Eqs. (6), (8), (13), and (15) are divided by the vertical stress, the relationships between the maximum or minimum fluid pressure ratios and differential stress for the extensional veins in reverse and normal fault stress regimes can be drawn in the $\lambda$–$\Delta \sigma$ diagram (Fig. 3). The lines from Eqs. (6) and (8) indicate the lower limit of the fluid pressure ratio, whereas the lines in Eqs. (13) and (15), including ${P}^{*}$, represent the upper bound of the fluid pressure ratio (Fig. 3). However, the vertical stress and rock tensile strength are still not constrained.

**Figure 4:**
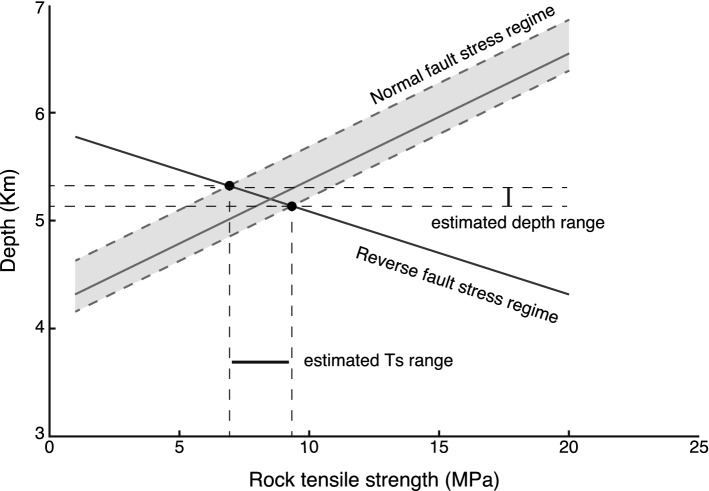
Relationship between the rock tensile strength and depth for normal and reverse fault stress regimes. See the text for the conversion from vertical stress to depth.

In order to constrain these parameters, we used previous estimates of fluid pressure based on fluid inclusion analysis from the network veins, which estimated that the fluid pressure was between 107(+ 8/− 4.1)–149.4 MPa^18^. As the previous study did not classify the extensional veins based on stress regime, the range of fluid pressures from the fluid inclusion was expected to be included in different stress regimes because the extensional veins were collected randomly in the previous study. When the differential stress is the maximum value of ${4T}_{s}$ (see the “Methods” section), the fluid pressure of 149.4 MPa must be the maximum in the reverse fault stress regime at point A in Fig. 3. However, the minimum fluid pressure of 107(+ 8/− 4.1) MPa was in the normal fault stress regime at point B, as shown in Fig. 3. When ${T}_{s}$ varies from 1 to 20 as reasonable values^33,34^, and using Eqs. (7) and (16), the relationships between ${T}_{s}$ and ${\sigma }_{v}$ at points A and B are constrained by the maximum differential stresses, $\Delta \sigma ={4T}_{s}$(Fig. 4). Vertical stresses are calculated using ${\sigma }_{v}=\rho gz$ (where $\rho$ is the rock density, $g$ is the gravitational acceleration, and $z$ represents depth), meaning that ${\sigma }_{v}$ can be converted to a depth ($\rho$ is 2.600 kg/m^3^ and $g$ is 9.8 m/s^2^ in the conversion). The cross-cutting relationship in the network veins suggests that the extensional veins formed at similar depths and rock tensile strengths in the normal and reverse fault stress regimes. Therefore, the cross point of the two slopes in the relationships between ${T}_{s}$ and depth for the normal and reverse fault regimes indicates the condition for extensional vein formation (Fig. 4). Consequently, ${T}_{s}$ and depth were constrained to 6.94–9.38 MPa and 5.14–5.33 km, respectively. In addition, the geothermal gradient was estimated to be approximately 24.39–29.17 °C/km based on the maximum paleo-temperature in the lower section of Mugi Mélange, which ranges from 130 to 150 °C^19^. The constrained values for tensile strength, depth and geothermal gradient are reasonable, which supports our expectation that extensional veins include both reverse and normal fault stress regimes.

### Quantifications of dynamic change in fluid pressure along a seismogenic fault

The difference between the lower and upper limits for fluid pressure in the $\lambda$–$\Delta \sigma$ diagram is $\Delta \sigma {P}^{*}$, and the maximum value of the difference is ${4T}_{s}{P}^{*}$, as shown by the differences between Eqs. (6) and (13) for the normal fault stress regime as well as Eqs. (8) and (15) for the reverse fault stress regime. The maximum differences are quantified to be 6.7–9.0 MPa for the normal fault stress regime and 8.3–11.3 MPa for the reverse fault stress regime when we use the constrained ${T}_{s}$ described above.

The absolute value of the fluid pressure ratio was also estimated using the constrained ${\sigma }_{v}$. The fluid pressure ratio in the reverse fault stress regime was approximately 1.12 at the maximum point A (Fig. 3), which exceeded the lithostatic pressure ($\lambda >1$). This is a requirement for an extensional vein in the reverse fault stress regime, as shown in Eqs. (8) and (15). Because the condition with $\lambda >1$ is unstable, we can interpret that a large fluid pressure was achieved in dynamic events. Previous studies have shown that the studied fault zones include geological evidence of thermal pressurization, which promoted seismic slip in this fault zone^24–26^. In our study, the dynamic fluid pressure reaching an over-lithostatic pressure indicated thermal pressurization during a seismic event. Our quantitative constraints indicate that fluid overpressures of about 6.7–11.3 MPa above the minimum requirement for the formation of extensional veins in a reverse fault stress regime were achieved at the time of the seismic event. The unstable condition with $\lambda >1$ also suggests that the fluid pressure increased by thermal pressurization, overcoming the dilatancy effect which tends to decrease the fluid pressure^35–37^.

However, our findings show that the fluid pressures in the reverse and normal fault stress regimes differ. The fluid pressure in the reverse fault stress regime was higher than that in the normal fault stress regime (Fig. 3). The smaller fluid pressure in the normal fault stress regime may indicate that fluid pressure decreases related to a fault-valve model, because the change in stress state between normal and reverse fault stress regimes can be related to seismic cycles, as discussed above. A decrease in fluid pressure may stabilize faults in a normal fault stress regime^35–37^. Our results also quantified the fluid pressure drop from the maximum to minimum fluid pressure after a seismic event, which was approximately 42 MPa from point A to point B in Fig. 3.

In our study, we constrained fluid pressure variations only in the extensional veins along a seismic fault, but we cannot rule out the possibility that the fluid pressure can decrease further after an earthquake to a level where extensional veins cannot form but instead promote the formation of shear veins^7,31^ when the differential stress becomes larger than ${4T}_{s}$; this means that the fluid pressure decrease can be larger than 42 MPa. It must be noted that the constrained fluid pressure range in this study applies only to the extensional veins, as was also true in a previous study^13^. Shear veins were observed close to the shear zone within the mélange in this study area, although the relationship between the shear veins in the mélange and the shear zone below the mélange needs to be examined carefully.

In summary we proposed new methods to constrain the depth, rock tensile strength, and dynamic fluid pressure variations from geological data. Our study successfully constrained the dynamic change in fluid pressure quantitatively in a seismic cycle, both during the increase stage caused by thermal pressurization and during the decrease stage caused by fault-valve behavior in a seismogenic fault zone. To fully evaluate fluid pressure fluctuations in seismogenic fault zones, investigations on other modes of failure, such as shear veins and hybrid extensional shear veins, must be expanded^7,31,32^.

## Methods

### Estimations of paleo-stress state and driving fluid pressure

We employed mixed Bingham statistics for a paleo-stress inversion from extensional veins^15,16^. The software GArcmB^16^ (http://bs.kueps.kyoto-u.ac.jp/tsg/software/GArcmB/) was used for the mixed Bingham statistics. The software fits the dataset with Bingham distributions and statistically identifies multiple Bingham distributions when multiple stress states are mixed in the network veins. Each Bingham distribution provides the principal stress orientations, and concentration parameters, ${\kappa }_{1}$, and ${\kappa }_{2}$^15,16^ (Supplementary Table S1). We can approximate stress ratio (Φ) by the ratio of the concentration parameters, $\Phi ={\kappa }_{2}/{\kappa }_{1}$^38^.

The stress ratio is defined as follows:

1 $$\Phi =\frac{{\sigma }_{2}-{\sigma }_{3}}{{\sigma }_{1}-{\sigma }_{3}},$$

where ${\sigma }_{1}$, ${\sigma }_{2}$, and ${\sigma }_{3}$ are the maximum, intermediate, and minimum principal stresses, respectively. The number of stress states (K) was set by the user as 1–5. The minimal value of the Bayesian information criterion (BIC) for each result with variables K was used to determine the optimal number of stress states in the mixed dataset^15,16^. Consequently, the optimal number of stress states in our dataset was three (Supplementary Fig. S2). The method also classifies the extensional veins that fit each stress state. The fitness of each extensional vein to a stress state is expressed by “membership”^15^. The membership indicates the probability that each extensional vein belongs to a stress state, ranging from 0 to 1. For each stress, the normal stresses normalized by the differential stress were calculated on each extensional vein. With the likelihood, the membership decreases as normalized normal stress increases. A previous study^39^ proposed the 95th percentile point of the probability, termed the driving pressure index (DPI), for the driving pressure ratio (${P}^{*}$) under fluctuated pressure conditions. DPI is defined as,

2 $$\frac{\underset{0}{\overset{DPI}{\int }}exp\left(-\left|{\kappa }_{1}\right|\sigma \right)d\sigma }{\underset{0}{\overset{\infty }{\int }}exp\left(-\left|{\kappa }_{1}\right|\sigma \right)d\sigma }=0.95,$$

where ${\kappa }_{1}$ and $\sigma$ are the concentration parameter and normalized normal stress.

Solving Eq. (2), we obtain

3 $$DPI\approx 3.00/\left|{\kappa }_{1}\right|.$$

We can use DPI as a representative value of ${P}^{*}$ for the Bingham distribution model^39^. ${P}^{*}$ is defined as^13^,

4 $${P}^{*}=\frac{{P}_{f}-{\sigma }_{3}-{T}_{s}}{{\sigma }_{1}-{\sigma }_{3}},$$

where ${P}_{f}$ and ${T}_{s}$ are fluid pressure and rock tensile strength. As three stress states were estimated from our dataset in this study, three ${P}^{*}$ were obtained for each stress state. However, in this study, we focused on two stress states for the normal and reverse fault stress regimes. The ${P}^{*}$ contains the lower bound of the maximum fluid pressure for the extensional vein formation^38^.

### Constraints on fluid pressure using rock failure theory

Brittle-rock failure is controlled by coefficients of internal friction, differential stress, and rock tensile strength (${T}_{s}$)^17,40,41^. Extensional cracks formed under a low differential stress of $\Delta \sigma <{4T}_{s}$^17^. Extensional veins are formed when the fluid pressure exceeds the sum of the normal stress on the extensional vein (minimum principal stress) and ${T}_{s}$^17,42^. Hence, extensional veins were formed when

5 $${P}_{f}>{\sigma }_{3}+{T}_{s}.$$

In the case of the normal fault stress regime, the maximum principal stress is equal to the vertical stress. Therefore, the minimum fluid pressure and vertical stress during the extensional vein formation under a normal fault stress regime can be approximated as follows:

6 $${P}_{f\_normal}={\sigma }_{v}-\Delta \sigma +{T}_{s},$$

where ${\sigma }_{v}$ and $\Delta \sigma$ are vertical stress and differential stress. Therefore,

7 $${\sigma }_{v}={P}_{f\_nomal}-{T}_{s}+\Delta \sigma .$$

Because the minimum principal stress in a reverse fault stress regime is equal to the vertical stress, the fluid pressure during extensional vein formation can be expressed as

8 $${P}_{f\_reverse}={\sigma }_{v}+{T}_{s},$$

9 $${\sigma }_{v}={P}_{f\_reverse}-{T}_{s}.$$

The fluid overpressure ($\Delta {P}_{O}$) is the excess fluid pressure greater than the minimum fluid pressure for the extensional veins, which is the sum of the minimum principal stress and rock tensile strength from Eq. (5). The fluid overpressure^13^ is defined as:

10 $$\Delta {P}_{O}=\left\{{P}_{f}-\left({\sigma }_{3}+{T}_{s}\right)\right\}={P}_{f}-{\sigma }_{3}-{T}_{s}.$$

The driving fluid pressure ratio (${P}^{*}$) is defined in Eq. (4), and it is rewritten as the $\Delta {P}_{O}$ normalized by $\Delta \sigma$^13^ as follows:

11 $${P}^{*}=\frac{{\Delta P}_{o}}{\Delta \sigma }=\frac{\left({P}_{f}-{\upsigma }_{3}-{T}_{s}\right)}{\left({\sigma }_{1}-{\sigma }_{3}\right)}.$$

We can thus express ${P}_{f}$ arranging Eq. (11) as follows

12 $${P}_{f}={\sigma }_{3}+{T}_{s}+{\Delta \sigma P}^{*}.$$

In the case of a normal fault stress regime, the minimum principal stress can be substituted with ${\sigma }_{v}-\Delta \sigma$. Therefore, in the normal fault stress regime, the fluid pressure and vertical stress can be calculated using Eq. (12), as follows:

13 $${P}_{f\_normal}={\sigma }_{v}+{T}_{s}-\left(1-{P}^{*}\right)\Delta \sigma ,$$

14 $${\sigma }_{v}={P}_{f\_normal}-{T}_{s}+\left(1-{P}^{*}\right)\Delta \sigma .$$

In a reverse fault stress regime, because the minimum principal stress is equal to the vertical stress, the fluid pressure during extensional vein formation can be expressed using Eq. (12) as

15 $${P}_{f\_reverse}={\sigma }_{v}+{T}_{s}+{\Delta \sigma P}^{*},$$

16 $${\sigma }_{v}={P}_{f\_reverse}-{T}_{s}-{\Delta \sigma P}^{*}.$$

## Supplementary Information

Supplementary Information.

## Data Availability

The data supporting the findings of this study are available in Supplementary information.
