# Supplementary material for: Geological constraints on dynamic changes of fluid pressure in seismic cycles
Source: Sci Rep. 2022 Aug 30;12:14789. doi: 10.1038/s41598-022-19083-x (PMC9427937; doi:10.1038/s41598-022-19083-x)
Supplement: Supplementary file 1 — Supplementary Information. [file 41598_2022_19083_MOESM1_ESM.pdf]

Supplementary information for

Geological constraints on dynamic changes of fluid pressure in seismic cycles

Takahiro Hosokawa[*1] and Yoshitaka Hashimoto[1]

[1] Department of Global Environment and Disaster Prevention, Faculty of Science and Technology, Kochi University, Akebonocho 2-5-1, Kochi 780-8520, Japan

[*]**Corresponding author**:

Takahiro Hosokawa (b22m6g61@s.kochi-u.ac.jp)

Department of Global Environment and Disaster Prevention, Faculty of Science and Technology, Kochi University, Akebonocho 2-5-1, Kochi 780-8520, Japan

Contents of this file

Figure S1, Figure S2, Table S1, and Table S2

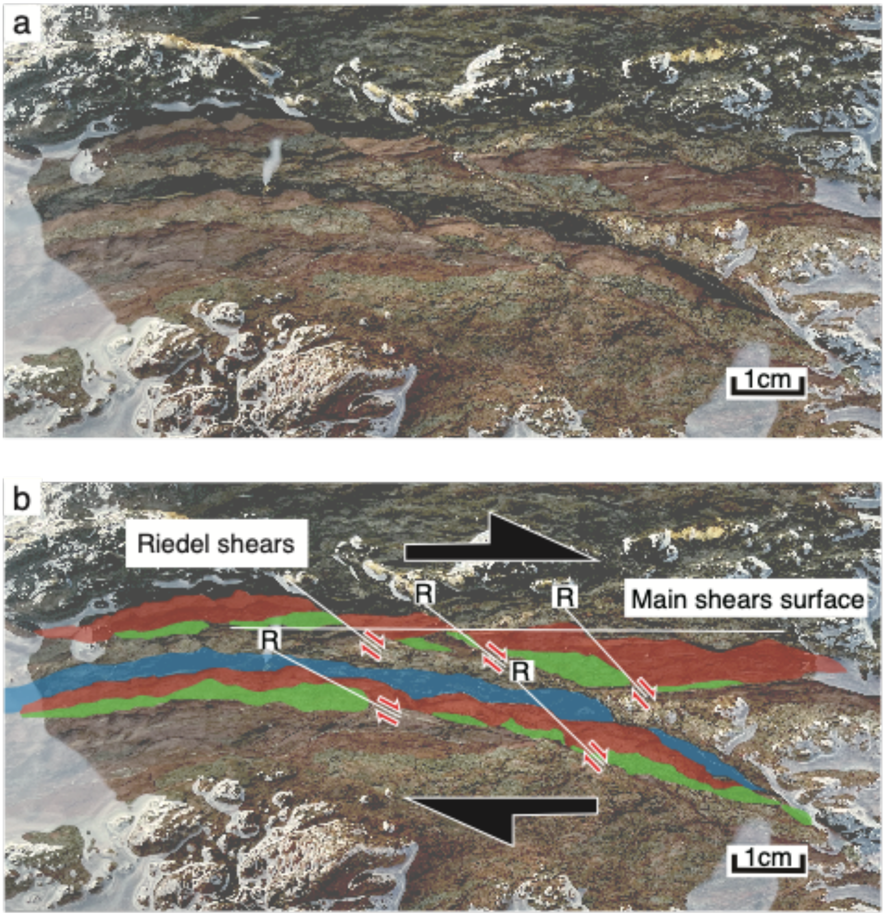

Figure S1: Riedel shears (R) in the shear zone without interpretations (a) and with interpretations (b) showing dextral shear deformation.

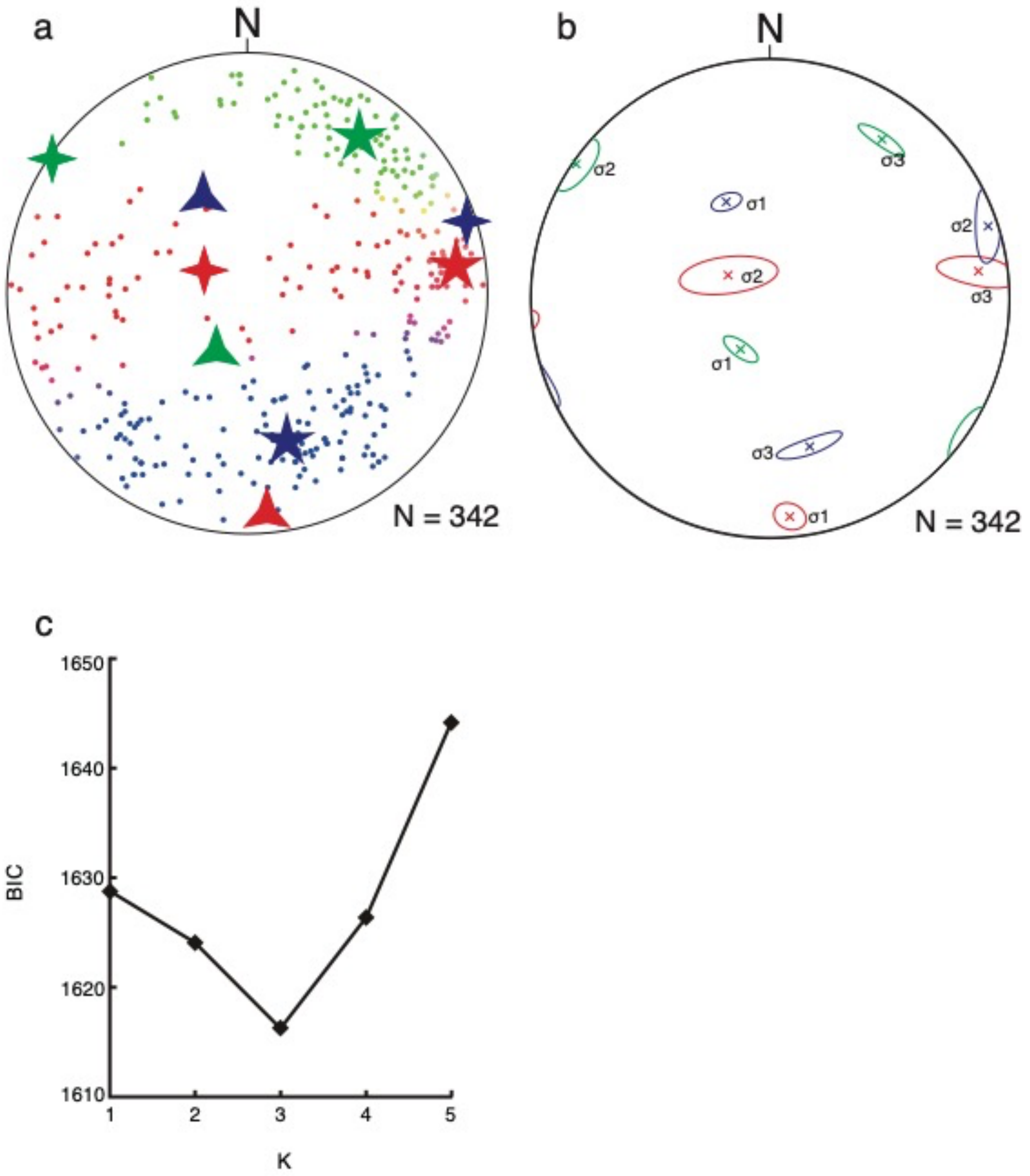

Figure S2: a) The orientations of the principal stress axes at the current setting before the rotation shown in a lower hemi-sphere equal area stereonet. Triangles, diamonds, and stars indicate the maximum, intermediate, and minimum principal axes. Stress 1, Stress

2 and Stress 3 are represented by the red, green and blue colors. The poles of the extensional veins are plotted by dots in the same stereonet. The dots indicate the extensional veins belonging to the Stress 1, Stress 2, and Stress 3 with the same color. b) 95 % confidence ellipse for each stress principal axis are represented by colored ellipses. c) A plot for BIC versus K. The minimum BIC at K=3 indicates that the optimal number of stress states is three.

Table S1: Results of estimated the principal stress orientations , concentration parameters, and stress ratio

Table S2: Strike and dip of extensional veins

Table S1. Results of estimated the principal stress orientations , concentration parameters, and stress ratio

	σ_1	σ_2	σ_3	κ_1	κ_2	Φ
Stress1	174.9º/9.2º	298.5º/73.7º	82.7º/13.4º	-10.044	-1.005	0.1000
Stress2	210.2º/69.8º	304.6º/1.6º	35.2º/20.1º	-20.585	-3.181	0.1545
Stress3	335.4º/53.6º	71.7º/4.6º	165.1º/36.0º	-12.402	-1.601	0.1291

Each principal stress axis is expressed by dip azimuth/dip angle.

Table S2 strike and dip of extension veins

250	52
200	52
88	68
226	38
269	52
262	42
218	38
238	52
212	14
92	58
114	34
114	48
222	22
246	42
235	58
91	84
89	48
254	32
156	32
154	32
96	48
78	78
18	62
134	52
255	30
190	60
319	55
325	60
340	60
220	75
250	65
240	72
322	50
245	70
335	50
310	50
79	72
238	80
259	72
350	52
258	82
220	86
198	82
210	84
332	58
208	62
226	74
198	68
218	66
216	72
218	62
204	70
190	64

208	78
226	68
188	72
212	60
210	72
206	66
198	62
216	56
210	56
194	64
222	58
238	62
204	78
208	80
234	74
172	68
220	72
228	64
230	68
152	78
232	82
192	84
178	76
228	78
46	62
324	78
18	80
186	70
282	70
150	72
332	72
222	82
322	78
322	74
236	68
324	72
260	72
32	78
234	78
234	78
32	78
330	68
314	50
220	84
212	84
158	68
222	76
232	72
238	80
194	76
268	60
202	74
172	70
70	80

2	64
352	60
222	72
202	64
200	72
26	68
338	62
320	78
280	72
38	68
206	78
302	54
238	82
228	66
344	54
156	78
244	74
268	72
176	68
258	72
32	72
342	52
198	62
332	60
262	82
216	80
32	72
322	54
28	70
336	60
312	58
332	60
12	66
70	78
240	70
318	68
232	58
58	62
284	70
268	66
252	72
32	60
310	64
316	60
338	60
92	64
266	54
306	52
230	50
262	72
260	78
262	70
128	52
258	38

134	34
110	50
250	14
278	74
98	38
272	32
262	80
94	48
92	42
250	48
86	78
266	68
92	88
118	30
108	56
268	30
104	58
100	80
264	18
262	80
338	48
280	60
312	52
286	58
190	78
328	40
322	32
46	88
238	80
272	60
320	44
302	40
320	32
292	62
358	68
348	60
350	50
80	68
60	50
230	76
40	68
284	72
40	78
176	80
178	80
46	60
244	82
260	84
268	80
48	72
178	80
344	50
228	72
310	62

358	36
356	30
2	32
72	82
356	22
32	40
8	72
10	58
232	68
356	48
40	62
260	54
352	46
330	46
48	68
250	60
42	70
200	80
38	56
332	58
342	58
34	72
248	58
42	68
252	66
318	50
42	62
264	72
48	58
268	68
60	74
198	58
302	48
344	62
30	76
304	50
140	70
78	44
318	54
80	46
280	58
112	74
230	60
340	56
342	60
260	66
242	62
50	78
60	80
320	54
328	62
54	60
356	58
22	58

40	60
26	40
284	58
340	38
82	78
296	66
38	48
10	48
342	38
346	38
34	82
74	56
352	80
278	58
272	12
22	84
352	86
262	52
82	46
158	70
340	72
256	82
310	20
212	68
6	84
344	68
346	38
58	16
240	78
20	38
290	40
66	66
264	66
260	68
272	80
290	60
284	68
258	68
110	82
302	68
264	70
342	66
344	62
352	42
262	52
248	82
258	58
314	68
250	88
260	42
247	23
256	79
261	80
268	88

313	48
8	61
8	12
282	74
54	86
330	68
322	70
346	72
104	76
18	68
358	16
340	52
32	84
354	70
156	88
40	58
66	78
288	46
262	76
